# New Books

**Published:** 2004-10

**America’s Environmental Report Card: Are We Making the Grade?**

Harvey Blatt

Cambridge, MA:MIT Press, 2004. 272 pp. ISBN 0-262-02572-8, $27.95

**Climate Change—Environment and Civilization in the Middle East**

Arie S. Issar, Mattanyah Zohar

London:Springer-Verlag, 2004. 252 pp. ISBN: 3-540-21086-5, $137.99

**Defying Ocean’s End: An Agenda for Action**

Linda K. Glover, Sylvia A. Earle, eds.

Washington, DC:Island Press, 2004. 250 pp. ISBN: 1-55963-753-6, $55

**Democracy’s Dilemma: Environment, Social Equity, and the Global Economy**

Robert C. Paehlke

Cambridge, MA:MIT Press, 2004. 316 pp. ISBN 0-262-66188-8, $16.95

**Diamond: A Struggle for Environmental Justice in Louisiana’s Chemical Corridor**

Steve Lerner

Cambridge, MA:MIT Press, 2004. 344 pp. ISBN 0-262-12273-1, $27.95

**Elements and their Compounds in the Environment: Occurrence, Analysis and Biological Relevance. 3 Volumes**

Ernest Merian, Manfred Anke, Milan Ihnat, Markus Stoeppler, eds.

Hoboken, NJ:John Wiley & Sons, 2004. 1,600 pp. ISBN: 3-527-30459-2, $680

**Encyclopedic Reference of Immunotoxicology**

Hans-Werner Vohr, ed.

London:Springer-Verlag, 2004. 1,000 pp. ISBN: 3-540-44172-7, $329.72

**Environmental Governance Reconsidered: Challenges, Choices, and Opportunities**

Robert F. Durant, Daniel J. Fiorino, Rosemary O’Leary, eds.

Cambridge, MA:MIT Press, 2004. 536 pp. ISBN: 0-262-54174-2, $35

**Environmental Impact Assessment of Recycled Wastes on Surface and Ground Waters: Chemodynamics, Toxicology, and Modeling Series**

Tarek A Kassim, Kenneth J. Williamson, eds.

London, England:Springer-Verlag, 2004. 450 pp. ISBN: 3-540-00268-5, $268.82

**Hope’s Horizon: Three Visions for Healing the American Land**

Chip Ward

Washington, DC:Island Press, 2004. 400 pp. ISBN: 1-55963-977-6, $27

**Implementing Climate and Global Change Research: A Review of the Final U.S. Climate Change Science Program Strategic Plan**

Committee to Review the U.S. Climate Change Science Program Strategic Plan, National Research Council

Washington, DC:National Academies Press, 2004. 108 pp. ISBN: 0-309-08865-8, $30

**Occupational Toxicology, 2nd Edition**

Neill H. Stacey, Chris Winder

Boca Raton, FL:CRC Press, 2004. 624 pp. ISBN: 0748409181, $89.95

**Pesticide Toxicology and International Regulation**

Timothy C. Marrs, Bryan Ballantyne, eds.

Hoboken, NJ:John Wiley & Sons, 2004. 592 pp. ISBN: 0-471-49644-8, $140

**Pharmaceuticals in the Environment: Sources, Fate, Effects and Risks, 2nd ed.**

Klaus Kümmerer, ed.

London:Springer-Verlag, 2004. 528 pp. ISBN: 3-540-21342-2, $110.20

**Protein Synthesis and Ribosome Structure: Translating the Genome**

Knud H. Nierhaus, Daniel N. Wilson, eds.

Hoboken, NJ:John Wiley & Sons, 2004. 592 pp. ISBN: 3-527-30638-2, $180

**Tutorials in Biostatistics, Volume 1—Statistical Methods in Clinical Studies**

Ralph B. D’Agostino

Hoboken, NJ:John Wiley & Sons, 2004. 350 pp. ISBN 0-470-02365-1, $150

**Tutorials in Biostatistics, Volume 2—Statistical Modeling of Complex Medical Data**

Ralph B. D’Agostino

Hoboken, NJ:John Wiley & Sons, 2004. 430 pp. ISBN 0-470-02370-8, $150

**Water Follies: Groundwater Pumping and the Fate of America’s Fresh Waters**

Robert Jerome Glennon

Washington, DC:Island Press, 2004. 304 pp. ISBN: 1-55963-400-6, $20

